# The mechanism of external cueing interventions in improving freezing of gait in Parkinson’s disease: an fNIRS study

**DOI:** 10.3389/fnagi.2026.1776882

**Published:** 2026-03-20

**Authors:** Ziyao Zhang, Lingyu Sheng, Guiyun Cui, Chunlei Shan, Jie Xiang

**Affiliations:** 1 Xuzhou Medical University, Xuzhou, China; 2 Xuzhou Medical University Affiliated Hospital, Xuzhou, China; 3 Rehabilitation Center, Tongren Hospital, Shanghai Jiao Tong University School of Medicine, Shanghai, China; 4 Yuanshen Rehabilitation Institute, Shanghai Jiao Tong University School of Medicine, Shanghai, China

**Keywords:** cortical activity, cue interventions, freezing of gait, functional connectivity, functional near-infrared spectroscopy, Parkinson’s disease, rhythmic auditory cues, rhythmic visual cues

## Abstract

**Introduction:**

External cue interventions can effectively improve gait disturbances in patients with Parkinson’s Disease (PD) and Freezing of Gait (FOG). However, the cortical mechanisms underlying cueing modulation of gait have rarely been investigated.

**Objectives:**

We aimed to compare gait performance and cerebral hemodynamic responses in patients with PD and FOG (PD-FOG) under different cueing interventions, and to further elucidate the neural mechanisms underlying the effects of different cues on gait by contrasting these findings with those of Healthy Controls (HCs).

**Methods:**

Twenty-eight PD-FOG patients and 28 HCs were enrolled. Gait parameters were measured during the walking experiment under Rhythmic Visual Cue (RVC) and Rhythmic Auditory Cue (RAC) to compare how these cues affect gait. Functional Near-infrared Spectroscopy (fNIRS) was employed to measure changes in oxyhemoglobin concentration (∆HbO_2_). Additionally, inter-channel connectivity strength was calculated to evaluate Functional Connectivity (FC) across various Regions of Interest (ROIs).

**Results:**

PD-FOG patients exhibit features including decreased gait velocity and stride length during freezing episodes. Both RVC and RAC enhance gait velocity and stride length. Conversely, the combined RVC and RAC (RVC + RAC) intervention did not produce meaningful changes in gait. Compared to HCs, PD-FOG patients show significantly lower ∆HbO_2_ in the Prefrontal Cortex (PFC) and Primary Somatosensory Cortex (S1). Both RVC and RAC interventions increase ∆HbO_2_ in the PFC and S1 in PD-FOG patients, whereas the RVC + RAC intervention decreases ∆HbO_2_ in the Premotor Cortex (PMC). Furthermore, compared with HCs, PD-FOG patients show increased FC between the S1-PFC and Primary Motor Cortex (M1)-PFC. The RVC intervention enhances FC within the PFC and between the PMC and the Visual Association Cortex (V2). The RAC intervention strengthens FC within the PFC and the PFC-Middle Temporal Gyrus (MTG). The RVC + RAC intervention increases FC within the PFC-MTG and PFC-PMC, partially compensating for functional deficits associated with cortical hypoactivation by enhancing connectivity among these ROIs.

**Conclusion:**

Single visual or auditory cues can improve FOG symptoms. RVC and RAC alleviate FOG by modulating cortical activation and enhancing FC between key ROIs. The heightened connectivity among these ROIs may represent the underlying neural pathway that mediates the cue-induced alleviation of FOG.

## 1 Introduction

Freezing of Gait (FOG) is one of the most disabling gait disorders in Parkinson’s Disease (PD), defined as “a brief, intermittent absence or marked reduction in forward foot movement despite the intention to walk” (Nutt et al., 2011). Up to 63% of PD patients experience FOG during their illness, with the probability rising to 90% in late stages (Cilia et al., 2015; Forsaa et al., 2015; Perez-Lloret et al., 2014). These episodes increase fall risk and significantly reduce quality of life (Kerr et al., 2010; Moore et al., 2007). However, FOG is often difficult to treat (Nutt et al., 2011; Vercruysse et al., 2014), and no effective pharmacological treatment currently exists. Recently, research on non-pharmacological treatments for FOG has grown. Alternative options—such as behavioral self-management strategies—have become an integral part of management (Rahman et al., 2008). Behavioral strategies are generally recommended to shift patients’ habitual movement control toward goal-directed movement control (Redgrave et al., 2010). Patients are taught to focus on gait using cue interventions (Lee et al., 2012) or cue combinations.

Cues are defined as discrete targets or references that trigger and facilitate movement execution (Nieuwboer et al., 2007). The primary neurophysiological mechanism behind external cues is the reorientation of neural circuits from affected to unaffected pathways (Redgrave et al., 2010). This shift indicates movement from habitual to goal-directed behavior. Interventions using external cues, such as visual and auditory stimuli, can effectively alleviate FOG symptoms in PD patients (Keus et al., 2007; Lee et al., 2012). Numerous studies report that rhythmic cues, in particular, modulate and improve FOG symptoms. These cues are widely recognized as effective therapeutic approaches for restoring gait function in patients with FOG (Lim et al., 2005; Lu et al., 2017; Nombela et al., 2013; Spaulding et al., 2013). Currently, rhythmic cues are categorized as Rhythmic Visual Cues (RVC) and Rhythmic Auditory Cues (RAC). Neuroimaging research shows that neural activity associated with rhythm perception is closely linked to motor regulation. Rhythm perception influences gait spatiotemporal parameters by activating impaired key motor networks in PD patients, such as the Premotor Cortex (PMC) and Supplementary Motor Area (SMA) (Nombela et al., 2013; Raglio, 2015). However, most studies focus on the effects of RVC, RAC, and combined RVC and RAC (RVC + RAC) intervention on gait improvement in FOG. There is no consensus on how rhythmic cues modulate cortical neural mechanisms during movement. To understand how the brain connects different cortical regions and how these regions interact, we use Functional Connectivity (FC) to reflect neural synchronization and coordinated activity across cortical areas (Sasai et al., 2012).

A cutting-edge advancement in neuroimaging—Functional Near-infrared Spectroscopy (fNIRS)—enables observation of hemodynamic activity within the cerebral cortex and functional connectivity between cortical regions (Ferrari and Quaresima, 2012). fNIRS produces low-motion artifacts and is portable, making it suitable for real-time monitoring of brain activity during movement. This non-invasive technique assesses changes in cortical oxygenated hemoglobin (ΔHbO_2_) and deoxygenated hemoglobin (∆HbR) by quantifying optical density differences between sources and detectors. Thus, it evaluates neurovascular coupling and cerebral hemodynamics (Pinti et al., 2020). Most fNIRS studies on PD patients’ brain activity during movement focus on the Prefrontal Cortex (PFC) (Belluscio et al., 2019; Maidan et al., 2015). Research confirms that PFC activation during walking influences gait performance and the walking process in PD patients (Maidan et al., 2016; Stuart et al., 2019). However, studies also show that the Primary Somatosensory Cortex (S1), Primary Motor Cortex (M1), and SMA are crucial for gait control (Harada et al., 2009; Koenraadt et al., 2014; Kurz et al., 2012). The mechanisms by which rhythmic cues modulate stepping patterns of patients with PD accompanied by FOG symptoms (PD-FOG) through these cortical regions, and the interconnections among relevant Regions of Interest (ROIs), remain unclear.

This study aims to investigate the effects of various rhythmic cues (RVC, RAC, and RVC + RAC intervention) on PD-FOG patients using fNIRS by exploring differences in cortical activation and FC associated with these cues. The goal is to elucidate the neural mechanisms underlying how different rhythmic stimuli influence FOG.

## 2 Methods

### 2.1 Participants

Based on the objectives of this study, we recruited patients with PD-FOG and age-matched Healthy Controls (HCs). Inclusion criteria for PD-FOG participants were: diagnosis of PD according to the 2015 Movement Disorder Society (MDS) clinical diagnostic criteria; age between 50 and 80 years; a score of ≥1 on item 3 of the Freezing of Gait Questionnaire (FOG-Q) to confirm FOG; Hoehn and Yahr (H-Y) stage II-III; ability to walk independently without assistive devices; stable medication for at least 4 weeks; sufficient hearing and vision (based on correct recall of the “whisper test” and 6/12 vision on the Snellen chart). All assessments were conducted while PD-FOG patients were in an “off-medication state,” including the Unified Parkinson’s Disease Rating Scale III (UPDRS III) (Goetz et al., 2008) and gait analysis. The “off-medication state” was achieved by withholding all anti-Parkinsonian medications (including levodopa and dopamine agonists) for at least 12 h overnight, with all evaluations performed in the morning before the patient’s first daily dose (Wang et al., 2024). The “off-medication state” was rigorously confirmed through a two-step process: (1) the patient’s last medication intake time; (2) verification of the onset of Parkinsonian motor symptoms by a clinical expert before the trial initiation. For HCs, eligibility required age between 50 and 80 years (matched to PD-FOG patients), independent walking ability, and adequate hearing and vision. Exclusion criteria for both PD-FOG and HCs included: cognitive impairment as measured by the Mini-Mental State Examination (MMSE) total score ≤ 24 (Nasreddine et al., 2012), other conditions affecting gait (e.g., musculoskeletal disorders), severe orthostatic hypotension, and movement limitations lasting more than 2 min. All participants provided written informed consent before participation. The study was conducted in accordance with the Declaration of Helsinki and was approved by the Ethics Committee of Xuzhou Medical University Affiliated Hospital (XYFY2025-KL351-01). It was part of a clinical trial registered on the Chinese Clinical Trial Registry (registration number: ChiCTR2500111409).

### 2.2 Gait assessments

The gait parameter data were recorded and analyzed using the Dynamic Gait and Posture Analysis System (Right Gait&Posture, Medical 3.0) developed by Shenzhen Xingzheng Technology Co., Ltd. This device comprises six pairs of pressure sensors in insoles of varying sizes and one pair of inertial sensors, operating at a sampling frequency of 100 Hz. The Dynamic Gait and Posture Analysis System consists of three layers: the sensor layer, the control layer, and the algorithm layer. The sensing layer primarily refers to sensor modules embedded within specialized insoles for both feet. These modules contain hundreds of pressure sensors capable of analyzing static and dynamic plantar pressure distribution. The inertial sensors integrate inertial measurement units, such as accelerometers and gyroscopes, with high-performance embedded microprocessors for signal detection. This enables real-time recording of spatiotemporal parameters during walking and kinematic characteristics of the ankle joint. Data collected is transmitted via Bluetooth 4.0 to the control layer, which then sends the data over the internet to the algorithm layer for cloud-based computation and data processing. Before the experiment, subjects select appropriate sensor-equipped insoles to place in their shoes (removing original insoles is recommended for comfort) and perform brief activities to adapt to the new insoles. Dynamic gait and posture analysis system data is compared with the Vicon three-dimensional motion capture system, showing 99.09% consistency in core metrics, including gait velocity, stride length, and cadence.

### 2.3 fNIRS examinations

**Figure 1:**
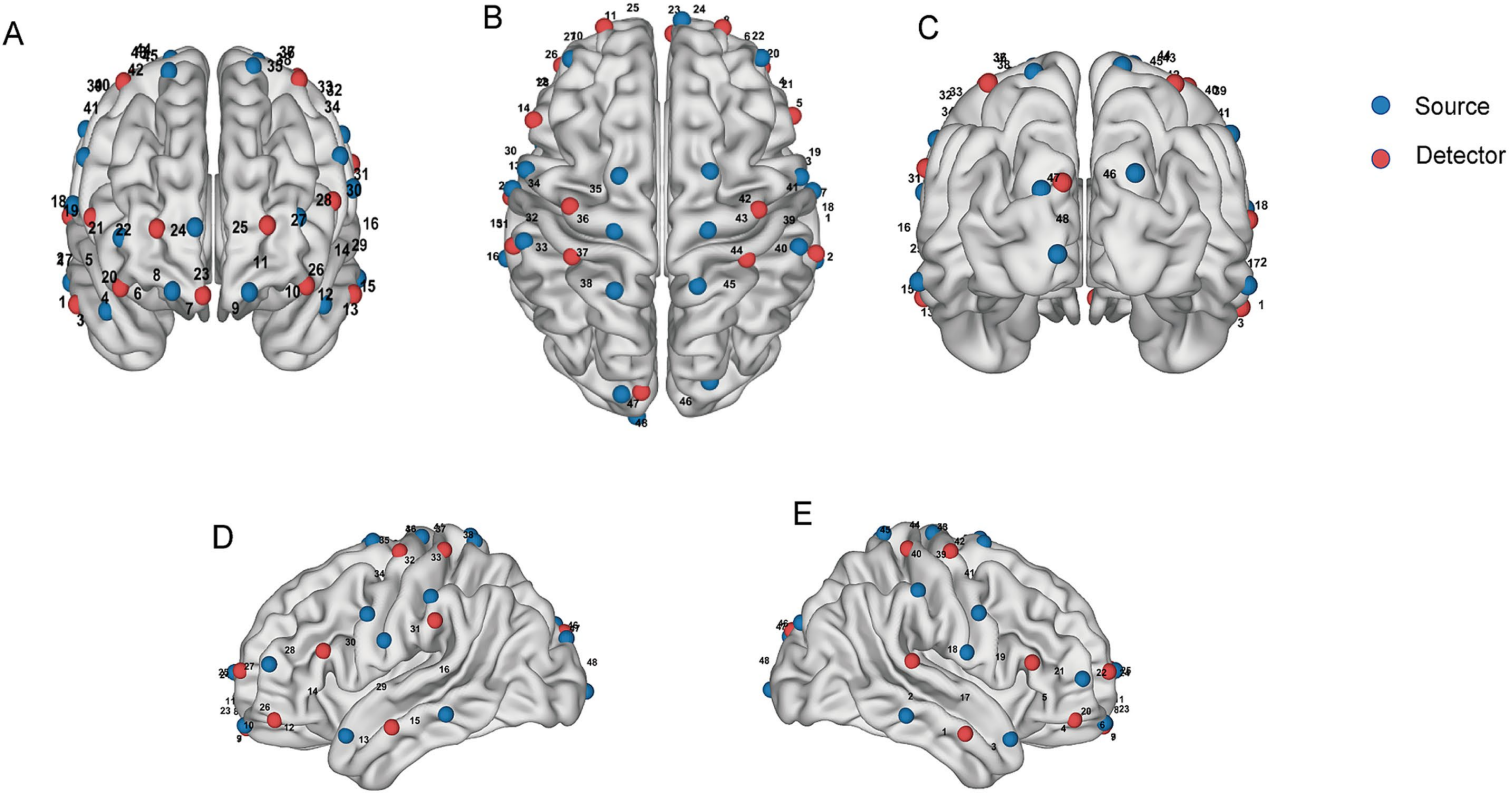
Schematic diagrams of the source and detector locations. **(A)** Front view. **(B)** Top view. **(C)** Rear view. **(D)** Left side view. **(E)** Right side view.

This experiment used the NirSmartII-3000A device (Danyang Huichuang Medical Equipment Co., Ltd., China) to measure and record ∆HbO_2_ and ∆HbR in the subjects’ brains during task performance. Light source probes used wavelengths of 730 nm and 850 nm. The sampling rate was 11 Hz. The setup included 24 light sources and 16 detectors, forming 48 effective channels (Figure 1). The average source-detector distance was 3 cm (range: 2.7–3.3 cm). Instrumentation followed three-dimensional positioning principles and the international 10–20 electrode placement system. Anatomical reference points were the nostrils, eardrums, and the anterior points of the left and right ears. This ensures the probe accurately covers the frontal, parietal, temporal, and occipital lobes. Channels were grouped into 9 ROIs based on their projection onto Brodmann Areas (BA): PFC, M1, S1, PMC, Somatosensory Association Cortex (SAC), Dorsolateral Prefrontal Cortex (DLPFC), Middle Temporal Gyrus (MTG), Primary Visual Cortex (V1), and Visual Association Cortex (V2). It is particularly important to note that although the DLPFC anatomically belongs to the PFC, channels covering BA9 and BA46 are considered DLPFC. In contrast, the remaining prefrontal channels (primarily covering the parietal pole of BA10 and BA11) are defined as PFC. This distinction is made to separate the executive control network from broader prefrontal functions clearly. This study independently defines the channel covering BA9 and BA46 as DLPFC, while the remaining prefrontal channels (primarily covering BA10 and BA11) are defined as PFC. This division ensures no spatial overlap between the ROIs.

The specific channels corresponding to each ROI are as follows: PFC (primarily corresponds to BA10/11): CH4, CH6, CH7, CH8, CH9, CH10, CH11, CH12, CH14, CH21, CH22, CH23, CH24, CH25, CH27, CH28; M1 (corresponds to BA4): CH37, CH44; S1 (corresponds to BA1/2/3): CH31, CH32, CH33, CH39, CH40; PMC (corresponds to BA6): CH19, CH30, CH34, CH35, CH36, CH41, CH42, CH43; SAC (corresponds to BA5/7): CH38, CH45; DLPFC (corresponds to BA9/46): CH20, CH26; MTG (corresponds to BA21): CH1, CH2, CH3, CH13, CH15, CH17; V1 (corresponds to BA17): CH48; V2 (corresponds to BA18): CH46, CH47.

### 2.4 Experimental procedures

**Figure 2:**
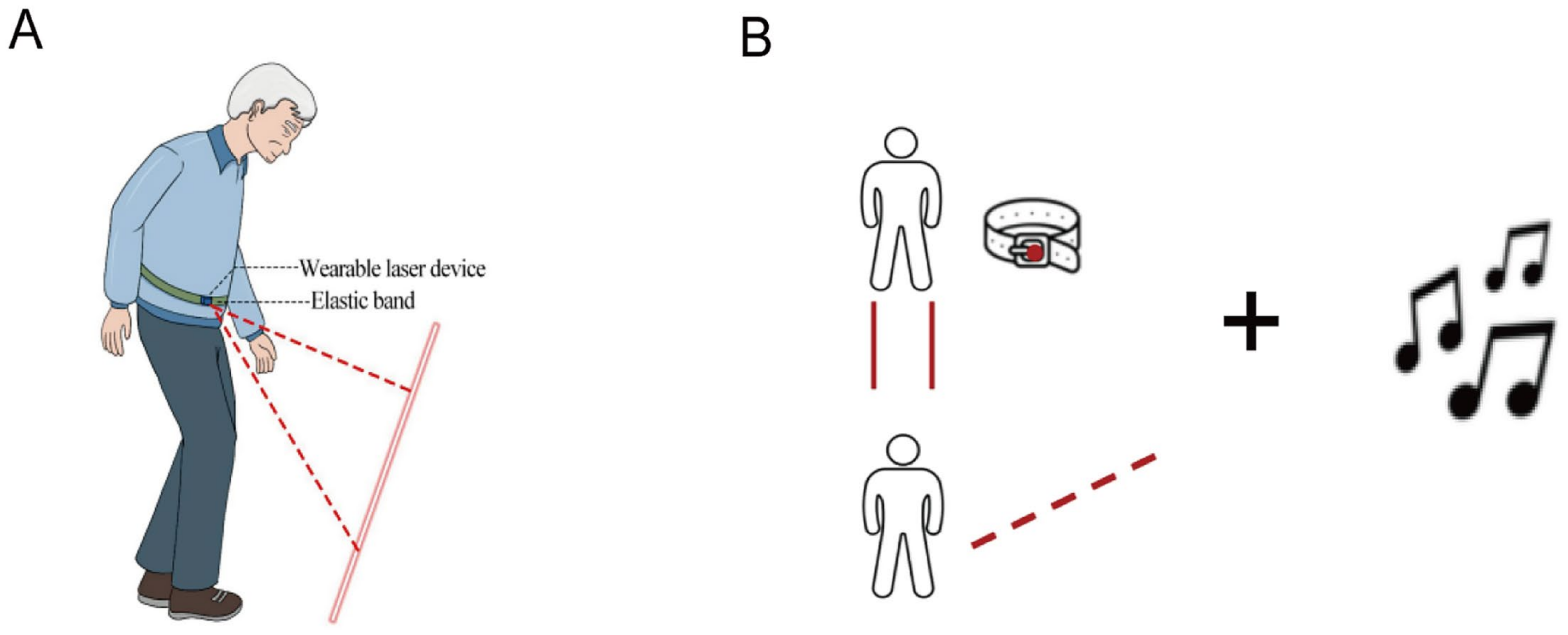
Rhythmic Visual Cue (RVC) intervention and RVC + RAC intervention **(A)** RVC intervention diagram: A wearable visual prompt device, fixed to an elastic cord worn around the waist, projects laser beams onto the ground to guide the subject along the beam. **(B)** RVC + RAC intervention diagram, the subject walks along the laser beam on the ground while listening to rhythmically strong music, with both interventions applied during the experiment.

This experiment must be conducted in quiet conditions, with participants independently wearing an fNIRS cap while performing walking tasks. For PD-FOG, four trials are conducted: No Intervention (NI) walking, RVC intervention walking, RAC intervention walking, and RVC + RAC intervention walking. The RVC utilized a wearable visual cueing device (Figure 2A) that projected a red laser line onto the ground. The laser line was 1 meter in length and 10 centimeters wide. Before the experiment began, the distance between the laser beam and the foremost point of each subject’s feet was set to 110% of each subject’s baseline stride length (Schlick et al., 2016). The baseline stride length was calculated from the average value obtained during a 10-meter walking test before the experiment. This distance was calibrated by adjusting the laser emitter’s projection angle before the experiment. It remained constant relative to the body during walking, requiring participants to lengthen their stride to step over the line. The RAC employed a piece of march music (“Back from Target Practice”) characterized by a prominent, high-intensity downbeat (2/4 time signature) to facilitate rhythmic entrainment (Figure 2B). The tempo (Beats per minute, BPM) of the RAC melody was set at 10% faster than the participant’s individual baseline cadence (Çarıkcı et al., 2021), which was measured in a 10-meter walking test before the experiment. BPM was modulated individually for each person’s cadence using digital audio processing software. The music was played through headphones, with the volume adjusted to a level the participant confirmed as clearly audible and comfortable, to ensure effective cue perception. The RVC + RAC intervention involved integrating both modalities, requiring participants to walk in sync with the musical rhythm while stepping along laser-guided lines. Before data collection, clinical researchers administered a brief cueing exercise to each participant, explicitly instructing them to synchronize their foot movements with visual cues and musical beats as closely as possible. This ensured participants could complete the experiment according to the cueing protocol. Throughout the formal experiment, clinical researchers closely monitored each experimental phase and participants’ engagement, ensuring that subjects remained focused on cue signals and adhered to task requirements. To minimize learning and fatigue effects, a Latin square design was employed to balance the sequence of four experimental conditions (NI, RVC, RAC, and RVC + RAC) among subjects. Mandatory five-minute rest intervals were implemented between each set of four walking experiments to allow subjects to recover to normal resting blood oxygen signals and prevent physical fatigue.

**Figure 3:**
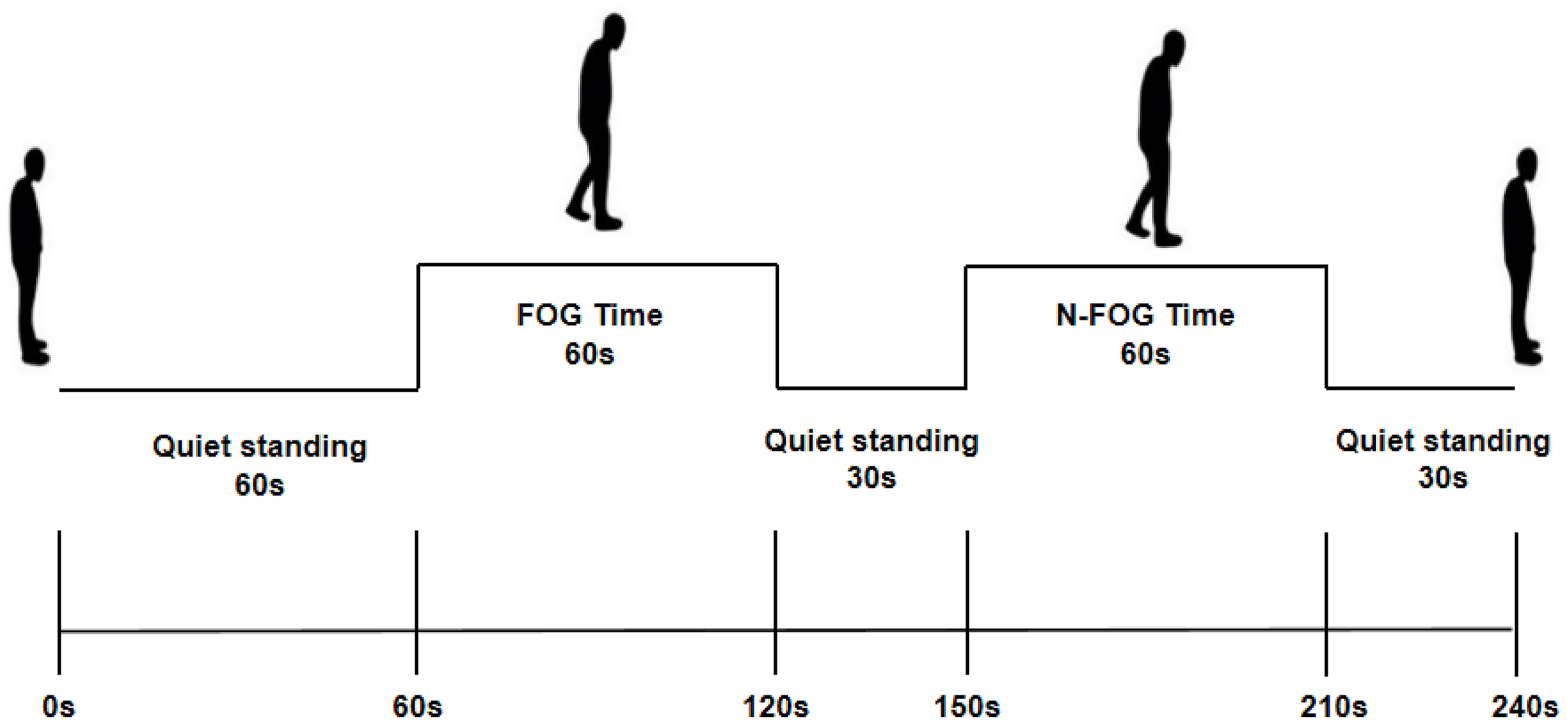
Experimental paradigm. The experiment lasted a total of 240 s, including 60 s of quiet standing, 60 s of freezing induction phase, 30 s of quiet standing, 60 s of freezing avoidance phase, and another 30 s of quiet standing. Freezing episodes were induced by turning in a narrow corridor, designated as the FOG Time. Subsequently, walking in a spacious area was performed to reduce the frequency of freezing episodes; this phase was labeled N-FOG Time.

The experimental paradigm comprised three components (Figure 3): 60 s of quiet standing, 60 s of continuous walking within a narrow corridor, followed by 30 s of quiet standing, and 60 s of walking in a spacious area, followed by 30 s of quiet standing. We designated the 60–120 s interval during the experiment as FOG Time. During FOG Time, participants were required to walk normally through the narrow passageway while also turning around at 75 s, 90s, and 105 s (once every 15 s, for a total of three turns). The freezing phenomenon was induced by walking through the narrow passageway and performing the three turns. Additionally, clinical professionals observed and recorded via video the number of FOG episodes, total FOG duration, and calculated the percentage of FOG time among the PD-FOG population during this time period. This ensured that the probability of freezing events occurring in subjects during the FOG Time was high. The 150–210 s interval was termed N-FOG Time, during which participants walked in an open space to prevent FOG occurrence. For HCs, a single walking trial without intervention is performed to compare cortical activity with that of the PD-FOG group.

### 2.5 Data analysis

#### 2.5.1 Preprocessing

We preprocessed the fNIRS data using the preprocessing module in NirSpark software (Danyang Huichuang Medical Equipment Co., Ltd., China), which had been employed in previous experiments (Li et al., 2023; Li et al., 2020). The preprocessing steps are as follows: First, all data undergo signal quality inspection. The coefficient of variation (CV) is used to calculate the signal-to-noise ratio (SNR) for each channel, and channels with CV > 15% are considered of low quality (Piper et al., 2014). Next, the standard deviation threshold is set to 6.0, and the amplitude threshold is set to 0.5. Motion artifacts are removed using a combination of standard deviation and cubic spline interpolation. Interference signals caused by heart rate, breathing rate, and Mayer waves were removed using a 0.01 to 0.2 Hz band-pass filter. Differential path-length factors were set to 6.0. Optical density was converted to blood oxygen concentration using the modified Beer–Lambert law (Li et al., 2025). From the preprocessed fNIRS data, the ∆HbO_2_ signal was used as the hemodynamic response indicator for further analysis, as ∆HbO_2_ is more sensitive to cerebral blood flow than ∆HbR (Bai et al., 2020). ∆HbO_2_ reflects cortical activation and correlates positively with activation intensity. The baseline was defined as the average blood oxygen saturation during the 5 s preceding the first step (the final 5 s of the quiet standing phase), aiming to capture the subject’s relative resting state and establish a local reference point closest to the start of the walking task. Given the inherent low-frequency drift characteristics of hemodynamic signals, this pre-task instantaneous baseline minimized the impact of extraneous physiological fluctuations and motion artifacts. Consequently, the relative change in HbO_2_ for each task condition was calculated by subtracting this baseline average from the mean HbO_2_ concentration during the task state. The ∆HbO_2_ signal was extracted from 2 to 60 s during walking (accounting for hemodynamic response latency: 1–2 s after neural discharge) (Balters et al., 2021).

#### 2.5.2 Functional connectivity

The FC matrix was computed in NirSpark using Pearson’s correlation coefficient for each channel pair. Fisher’s r-to-z transformation was conducted to improve normality. For each participant, a 48 × 48 correlation matrix was generated. The ROI-to-ROI and ROI-to-self correlation coefficients for each participant were calculated. The time series of all channel pairs was averaged for each participant, contributing to the participant’s overall FC.

### 2.6 Statistical analysis

Statistical analyses were conducted using SPSS version 27.0. Graphical representations were generated with NirSpark and GraphPad Prism 10. Before analysis, the Shapiro–Wilk test was employed to assess data normality, and Levene’s test was used to assess homogeneity of variances. Variables conforming to a normal distribution (age, body mass index, weight, educational level, MMSE) were analyzed using two-sample *t*-tests. For continuous variables that do not meet the normality and homogeneity of variance assumptions (e.g., height), the Mann–Whitney *U* test was used, with results reported as medians and interquartile ranges (Q25, Q75). Categorical variables, including gender and H-Y staging, were analyzed using chi-square tests. Given that the study involved repeated measures of the same participants under different conditions (no intervention, RVC, RAC, RVC + RAC intervention), one-way repeated measures ANOVA was employed to compare gait parameters (gait velocity, stride length, cadence) and fNIRS data (channel activation, ROI activation, channel FC, ROI-to-ROI FC). Effect sizes are represented by Cohen’s standardized mean difference (Cohen’s *d*) and partial eta-squared (partial *η^2^*). To control the false positive rate associated with multiple comparisons across multiple measurement channels, we implemented False Discovery Rate (FDR) correction using the Benjamini–Hochberg (BH) procedure (Benjamini and Hochberg, 1995). Specifically, *p*-values from one-way repeated measures ANOVA for each channel were ranked in ascending order (*p₁ ≤ p₂ ≤ … ≤ pₙ*), and the significance threshold was determined by *Pk ≤ α*, where *Pk = k/n × α* (*n*: total number of channels, *k*: the rank position in the sequence). Only channels passing this spatial FDR correction (*q* < 0.05) were considered to exhibit a significant main effect. For channels that passed the FDR correction, Fisher’s Least Significant Difference (LSD) test was subsequently performed for *post hoc* pairwise comparisons among cueing conditions. This hierarchical statistical strategy first restricts false positive rates at the spatial level (via FDR) while maintaining sufficient sensitivity to detect subtle variations across different conditions (avoiding Type II errors) (Genovese et al., 2002; Singh and Dan, 2006; Ye et al., 2009). All *p*-values reported are based on two-sided tests, with values less than 0.05 considered statistically significant.

## 3 Results

### 3.1 Characteristics of the participants

This study included 56 participants: 28 patients with PD exhibiting FOG symptoms and 28 age-matched HCs. As shown in Table 1, there were no significant differences between the PD-FOG and HCs in gender, age, height, weight, educational level, or MMSE scores. The disease duration, severity of freezing (as measured by the FOG-Q), and fall risk [predicted by Tinetti Performance-Oriented Mobility Assessment (POMA) scores] in PD-FOG patients were also included in Table 1. Additionally, Table 1 details the number of FOG episodes, total FOG duration, and the percentage of FOG time for PD-FOG patients during FOG Time.

**Table 1:** Comparison of general and clinical data among the PD-FOG NI group and HCs.

Basic information	PD-FOG (*n* = 28)	HCs (*n* = 28)	Statistical value	*p*-value
Age (years)	69.86 ± 6.26	67.71 ± 4.86	1.43	0.158
Gender (male/female)	20/8	13/15	3.615	0.102
Height (m)	1.65(1.57, 1.71)	1.67(1.58, 1.74)	0.296	0.767
Weight (kg)	67.96 ± 10.55	69.46 ± 10.71	0.528	0.600
Body Mass Index (kg/m^2^)	24.79 ± 3.00	25.00 ± 2.14	0.312	0.756
Educational level (years)	11.75 ± 1.69	11.89 ± 1.85	0.301	0.764
MMSE (0–30)	26.18 ± 1.39	25.50 ± 1.45	0.289	0.593
Disease duration (years)	6.93 ± 2.75	/	/	/
UPDRS-III: off (0–132)	37.11 ± 9.02	/	/	/
H-Y stage (II/III)	11/17	/	/	/
FOG-Q (0–24)	10.14 ± 2.22	/	/	/
POMA (0–28)	25.46 ± 1.60	/	/	/
Number of FOG episodes (times)	3.54 ± 1.53	/	/	/
Total FOG duration (seconds)	15.07 ± 7.48	/	/	/
Percentage of FOG time (%)	25.12 ± 12.46	/	/	/

PD-FOG, Parkinson’s disease patients with freezing of gait; HCs, healthy controls; H-Y, Hoehn–Yahr; FOG-Q, Freezing of Gait Questionnaire; MMSE, Mini-Mental State Examination; UPDRS-III, Unified Parkinson’s Disease Rating Scale Part III; POMA, Tinetti Performance-Oriented Mobility Assessment.

### 3.2 Gait performance

Compared to N-FOG Time, gait parameters in the PD-FOG NI group during FOG Time showed slower gait velocity (*t* = 4.733, *p* < 0.001, Cohen’s *d* = 0.890), shorter stride length (*t* = 4.090, *p* < 0.001, Cohen’s *d* = 0.770). Detailed gait characteristics are presented in Table 2. These findings indicate poorer gait performance and decreased stability during FOG Time, likely due to a higher frequency of freezing episodes caused by turning in narrow passages. No statistically significant differences in cadence were observed between FOG and N-FOG Time.

**Table 2:** Gait parameters of the PD-FOG NI group among FOG Time and N-FOG time.

Gait parameters	FOG Time	N-FOG Time	Statistical value	*p-*value	Cohen’s *d*
Stride length (cm)	50.14 ± 15.42	68.61 ± 25.71	4.090	<0.001	0.770
Gait velocity (m/s)	0.43 ± 0.21	0.63 ± 0.26	4.733	<0.001	0.890
Cadence (steps/min)	104.88 ± 18.11	111.74 ± 23.06	1.382	0.178	0.260

FOG Time, the period of 60–120 s in this experimental paradigm; N-FOG Time, the period of 150–210 s in this experimental paradigm; Cohen’s *d*, Cohen’s standardized mean difference.

**Figure 4:**
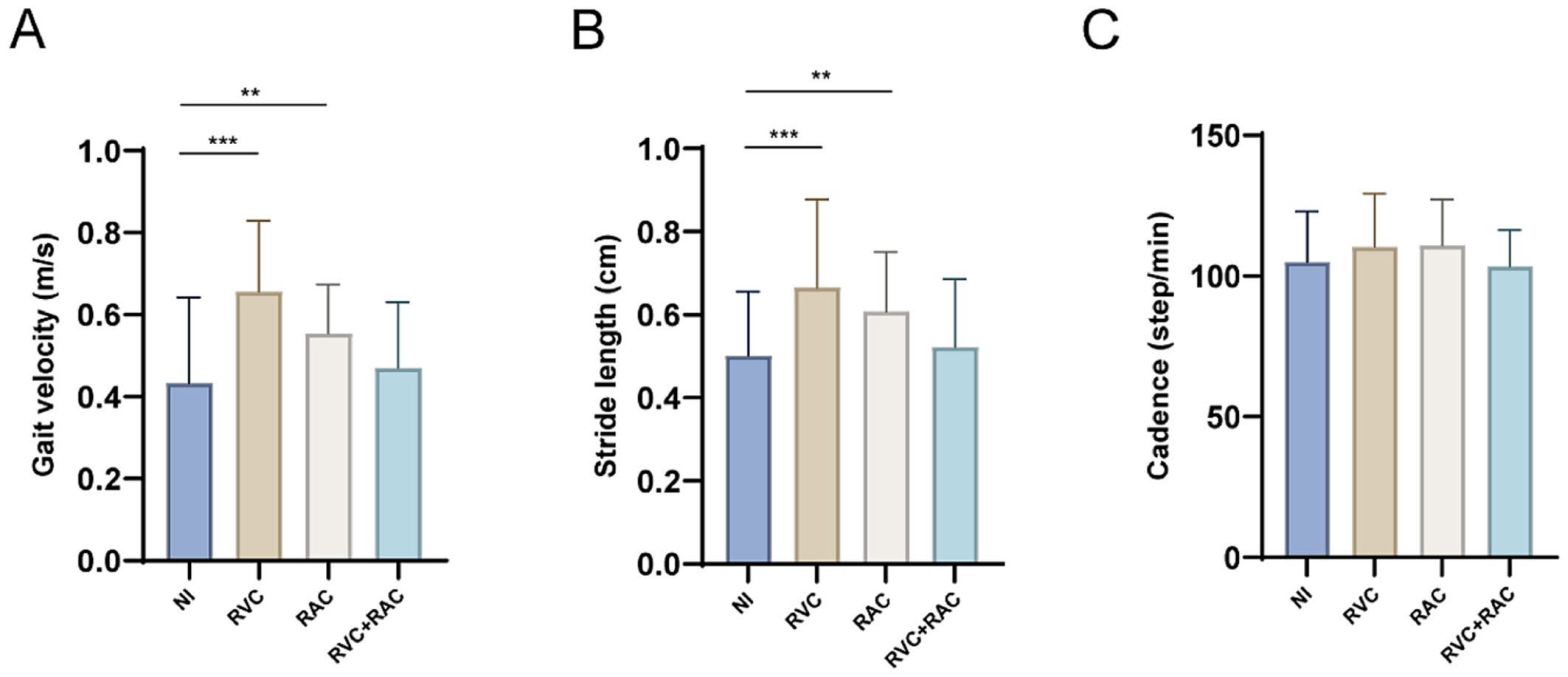
Gait parameters in PD-FOG four intervention groups during FOG Time. **(A)** Gait velocity: both RVC and RAC interventions significantly improved gait velocity compared to the NI group, with RVC showing a greater improvement. **(B)** Stride length: RVC and RAC interventions significantly increased stride length compared with the NI group, with RVC demonstrating a superior effect. **(C)** Cadence: no significant changes observed. **p < 0.01, ***p < 0.001.

Figure 4 shows that four intervention groups in PD-FOG (NI, RVC, RAC, and RVC + RAC) were applied during FOG Time. Comparative analysis of gait parameters among these groups revealed no statistically significant differences in cadence (*F* = 2.131, *p* = 0.111, partial *η^2^ =* 0.073). However, significant differences were observed in other gait metrics, including stride length (*F* = 7.185, *p* = 0.001, partial *η^2^ =* 0.210) and gait velocity (*F* = 12.987, *p* = 0.001, partial *η^2^ =* 0.325). Detailed data are provided in Supplementary Table 1. Both RVC (66.54 ± 21.28, *p* < 0.001) and RAC (60.79 ± 14.33, *p* = 0.003) conditions significantly increased stride length compared to NI (50.14 ± 15.42), whereas the RVC + RAC intervention did not produce a statistically significant effect on stride length (*p* = 0.694). RVC demonstrated greater improvement in stride length than RAC. Regarding gait velocity, both RVC (0.66 ± 0.17, *p* < 0.001) and RAC (0.55 ± 0.12, *p* = 0.005) significantly increased walking speed, but combining them did not yield additional benefits (*p* = 0.363). Notably, RVC was more effective than RAC in enhancing gait velocity. Overall, rhythmic visual and auditory cues significantly improved stride length and gait velocity, with RVC being more effective. Conversely, the combined RVC and RAC intervention did not produce significant improvements in gait parameters, such as stride length and gait velocity. This may be attributed to the limited attentional resources of PD-FOG patients, in which processing both visual and auditory cues impairs gait performance.

### 3.3 HbO_2_ concentration changes

#### 3.3.1 Brain activation in the PD-FOG NI group compared to HCs

**Figure 5:**
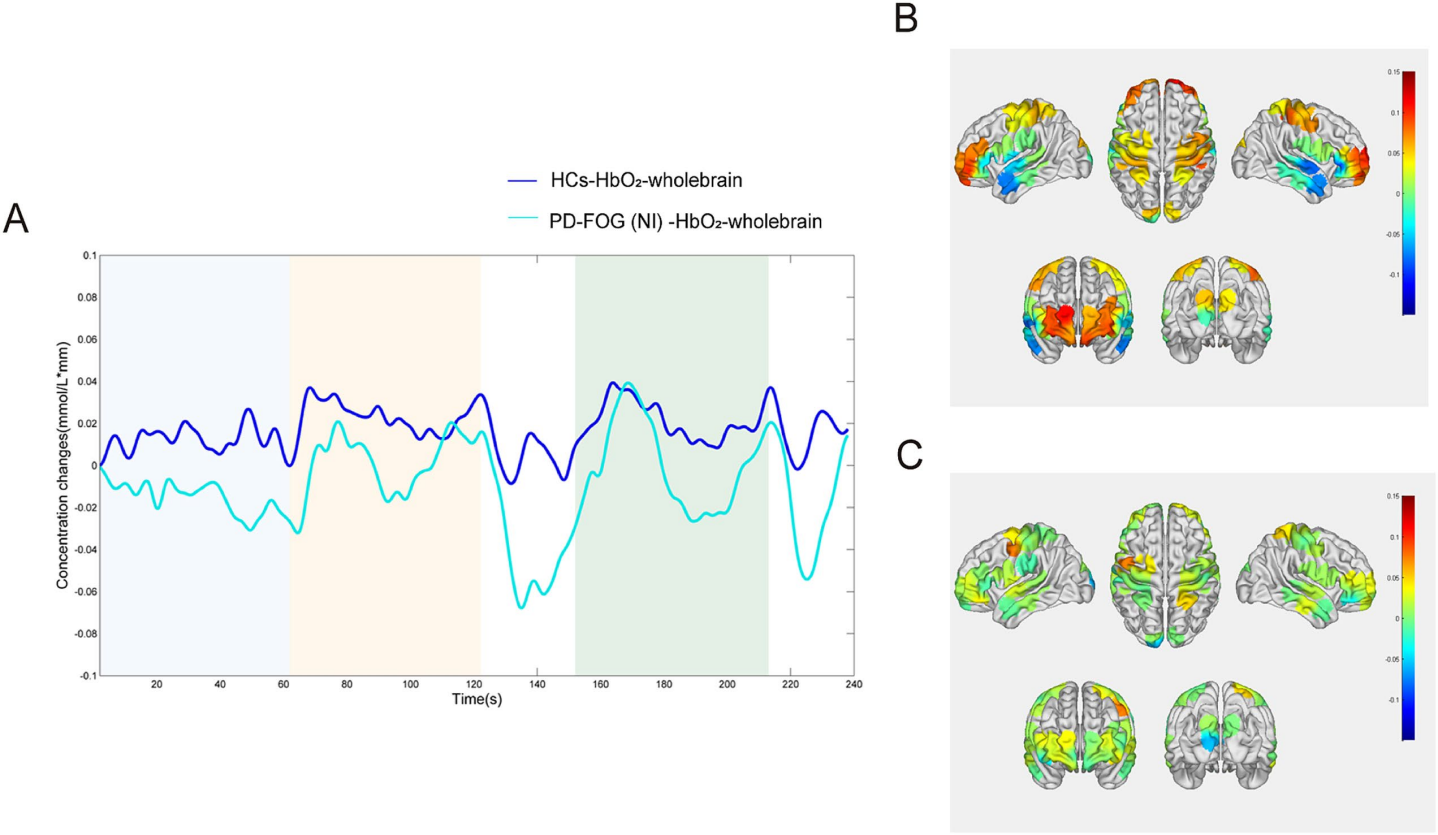
ΔHbO_2_ time courses and topographical maps of PD-FOG NI group and HCs across the entire duration. **(A)** ΔHbO_2_ time courses of PD-FOG NI group and HCs, with overall ΔHbO_2_ in PD-FOG NI group being lower than that in HCs. **(B)** Topographical map of ΔHbO_2_ in HCs, showing higher ΔHbO_2_ compared to the PD-FOG NI group **(C)**. Topographical map of ΔHbO_2_ in the PD-FOG NI group.

By plotting the overall ΔHbO_2_ time courses of the PD-FOG NI group and HCs across different time periods, we observed that the ∆HbO_2_ fluctuations in the HCs were generally higher than those in the PD-FOG NI group, as shown in Figure 5A. This was consistent across quiet standing time (blue segments), FOG time (yellow segments), and N-FOG Time (green segments). The ∆HbO_2_ in the PD-FOG NI group was markedly lower than in HCs. Additionally, Figure 5A shows that HCs exhibited relatively stable fluctuations in blood oxygen levels overall. In contrast, the PD-FOG group experienced rapid fluctuations in blood oxygen levels across different time periods (e.g., at the end of the FOG Time). It reached their lowest HbO_2_ levels earlier than HCs during the FOG Time. Collectively, these findings indicated that the PD-FOG group exhibited more unstable HbO_2_ fluctuations in the cerebral cortex, with a greater tendency toward rapid fluctuations and greater amplitude of variation compared to healthy individuals. Figures 5B,C present the brain activation topography during FOG Time for both groups. Regions with more intense activation are indicated by closer proximity to red, while less active areas are closer to blue. The diagram clearly shows that the HCs exhibited greater cortical activation, particularly in the prefrontal and parietal regions.

Based on ROI analysis, we re-evaluated differences in brain activation during FOG Time and summarized the results in Table 3. Significant differences in ∆HbO_2_ were observed in the S1 (*t* = 3.274, *p* = 0.002, Cohen’s *d* = 0.833), M1 (*t* = 3.115, *p* = 0.003, Cohen’s *d* = 0.767), and PFC (*t* = 2.754, *p* = 0.008, Cohen’s *d* = 0.658) regions between PD-FOG patients and HCs during FOG Time. No significant differences were found in the PMC and SAC regions. Notably, most ∆HbO_2_ in the HCs were positive, whereas in the PD-FOG NI group, they were predominantly negative, indicating that ∆HbO_2_ in S1, M1, and PFC were significantly lower in PD-FOG patients during FOG Time, consistent with the waveform and topographical imaging results.

**Table 3:** ∆HbO_2_ ((mmol/L)*mm) of the PD-FOG NI group and HCs among different ROIs in FOG time.

Time	ROIs	PD-FOG (*n* = 28)	HCs (*n* = 28)	Statistical value	*p-*value	Cohen’s *d*
FOG Time	S1	−0.01 ± 0.06	0.04 ± 0.06	3.274	0.002	0.833
	M1	−0.01 ± 0.07	0.04 ± 0.06	3.115	0.003	0.767
	PFC	−0.01 ± 0.07	0.03 ± 0.05	2.754	0.008	0.658
	PMC	0.01 ± 0.05	0.02 ± 0.04	0.825	0.413	0.221
	MTG	0.02 ± 0.08	−0.00 ± 0.08	1.119	0.268	0.250
	SAC	0.01 ± 0.05	0.03 ± 0.05	1.254	0.215	0.400
	DLPFC	−0.01 ± 0.08	0.03 ± 0.06	1.643	0.106	0.566

ROIs, regions of interest; S1, primary somatosensory cortex; M1, primary motor cortex; PFC, prefrontal cortex; PMC, premotor cortex; MTG, middle temporal gyrus; SAC, somatosensory association cortex; DLPFC, dorsolateral prefrontal cortex; Cohen’s *d* values are presented as absolute values.

#### 3.3.2 Brain activation differences among the four PD-FOG subgroups

**Figure 6:**
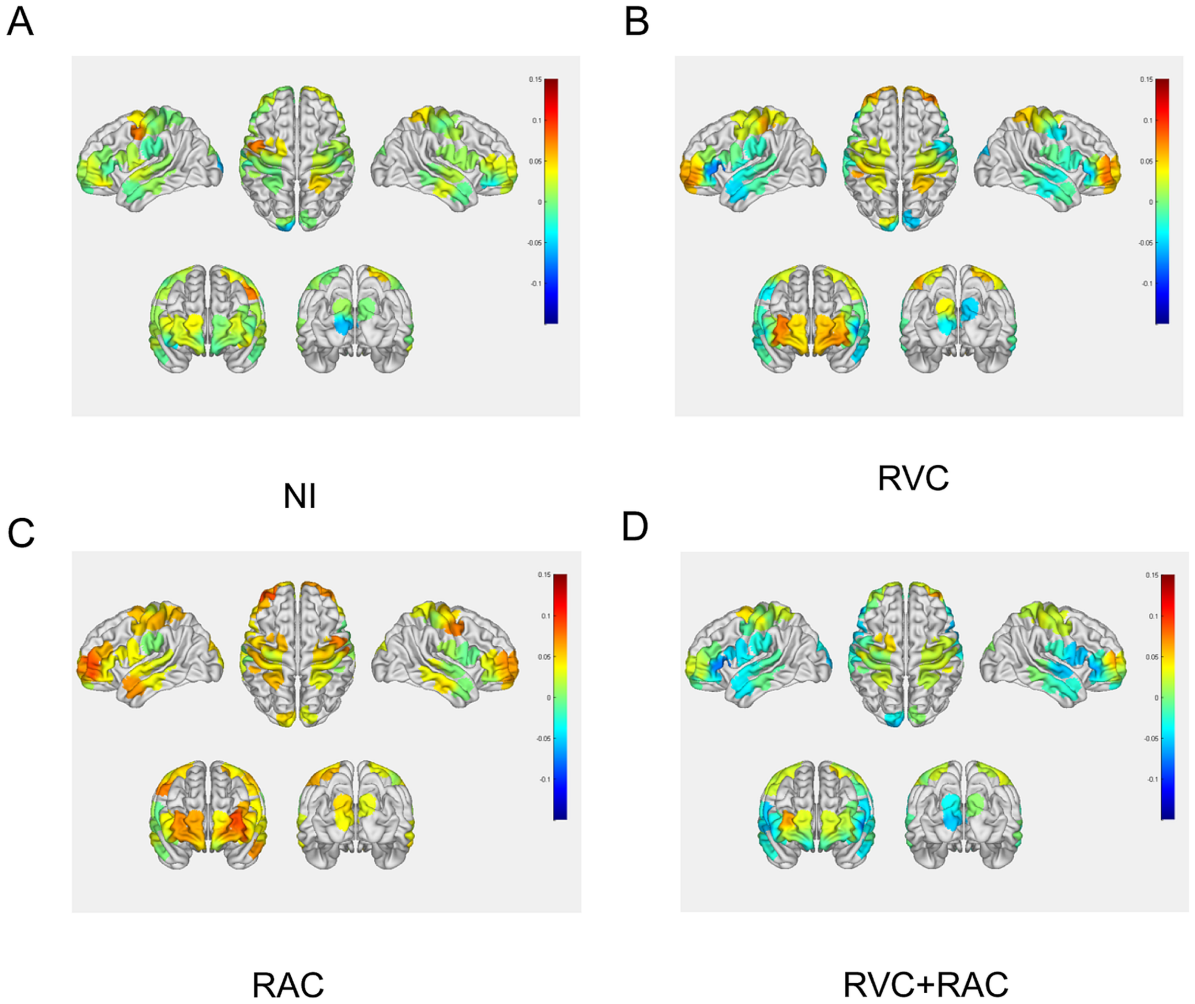
Brain activation topography maps of the four PD-FOG groups. **(A)** ΔHbO_2_ topography map of the PD-FOG NI group. **(B)** ΔHbO_2_ topography map of the PD-FOG group under RVC intervention. **(C)** ΔHbO_2_ topography map of the PD-FOG group under RAC intervention. **(D)** ΔHbO_2_ topography map of the PD-FOG group under RVC + RAC intervention.

First, the brain activation topography maps of the four PD-FOG groups were generated (Figure 6). The results indicate that both RVC and RAC interventions significantly increased ∆HbO_2_ compared to the NI group, with RAC demonstrating a superior effect. However, the combined intervention did not further enhance ∆HbO_2_ or promote additional neural activation. A one-way repeated measures ANOVA at the ROI level revealed no statistically significant differences among the four groups. Conversely, at the channel level, comparisons between the NI group during FOG Time and the three cue-based intervention groups showed significant differences in ∆HbO_2_ at the following channels (Table 4): CH19 (*F* = 3.579, *p* = 0.025, partial *η^2^ =* 0.117), CH23 (*F* = 3.264, *p* = 0.032, partial *η^2^ =* 0.108), CH25 (*F* = 3.663, *p* = 0.020, partial *η^2^ =* 0.137), CH27 (*F* = 3.617, *p* = 0.019, partial *η^2^ =* 0.118), and CH33 (*F* = 4.206, *p* = 0.015, partial *η^2^ =* 0.135).

**Table 4:** ∆HbO_2_ [(mmol/L)*mm] of the PD-FOG four intervention groups in FOG time.

Time	CH	NI	RVC	RAC	RVC+RAC	Statistical value	*p*-value	Partial *η^2^*	*p*-value (*post hoc*)		
									None vs. RVC	None vs. RAC	None vs. RVC+RAC
FOG Time	CH19	0.03 ± 0.09	0.01 ± 0.07	0.00 ± 0.08	−0.04 ± 0.07	3.579	0.025	0.117	0.362	0.119	0.004
	CH23	−0.02 ± 0.07	0.02 ± 0.07	0.03 ± 0.09	−0.02 ± 0.06	3.264	0.032	0.108	0.032	0.032	0.784
	CH25	−0.04 ± 0.09	0.03 ± 0.07	0.03 ± 0.10	0.00 ± 0.07	3.663	0.020	0.137	0.014	0.012	0.138
	CH27	0.00 ± 0.08	0.04 ± 0.08	0.06 ± 0.11	−0.01 ± 0.08	3.617	0.019	0.118	0.099	0.027	0.535
	CH33	0.00 ± 0.08	0.04 ± 0.05	0.03 ± 0.06	0.01 ± 0.06	4.206	0.015	0.135	0.019	0.018	0.468

CH, channel; NI, no intervention; RVC, rhythmic visual cue; RAC, rhythmic auditory cue; RVC+RAC, combined RVC and RAC intervention; partial *η*^2^, partial eta-squared.

For these meaningful channels, we classified the relevant pathways into corresponding ROIs based on Brodmann areas. As shown in Table 4, regarding the channels within PFC: CH23, CH25, and CH27, the ∆HbO_2_ indicated that RVC increased activation in CH23 (*p* = 0.032) and CH25 (*p* = 0.014), but had no significant effect on CH27 (*p* = 0.099). However, all three channels exhibited significant increases in ∆HbO_2_ under RAC intervention (CH23: *p* = 0.032; CH25: *p* = 0.012; CH27: *p* = 0.027). Conversely, combined RVC + RAC intervention did not produce significant changes in ∆HbO_2_ for CH23 (*p* = 0.784), CH25 (*p* = 0.138), or CH27 (*p* = 0.535). For the channel within S1: CH33, both RVC (*p* = 0.019) and RAC (*p* = 0.018) significantly increased ∆HbO_2_, whereas the combined RVC and RAC intervention showed no statistically significant effect (*p* = 0.468). Unlike the channels in PFC and S1, CH19, which belongs to PMC, demonstrated a significant effect only under the RVC + RAC intervention (*p* = 0.004). Notably, this intervention resulted in a marked decrease in ∆HbO_2_ in CH19, indicating that combined audio-visual cue stimulation is associated with PMC involvement in motor regulation and that this stimulation reduces PMC activation.

A comparison of the intervention effects across several cueing conditions revealed that in the PFC, RAC (0.03 ± 0.09, *p* = 0.032, partial *η^2^ =* 0.108) produced a slightly greater increase in ∆HbO_2_ for CH23 than RVC (0.02 ± 0.07, *p* = 0.032, partial *η^2^ =* 0.108). In CH25, the effects of both interventions were comparable (RAC: 0.03 ± 0.10, *p* = 0.012, partial *η^2^ =* 0.137; RVC: 0.03 ± 0.07, *p* = 0.014, partial *η^2^ =* 0.137). For CH27, only RAC intervention significantly elevated ∆HbO_2_ (*p* = 0.027, partial *η^2^ =* 0.118). In the S1 region of CH33, RVC (0.04 ± 0.05, *p* = 0.019, partial *η^2^ =* 0.135) demonstrated a marginally better enhancement of ∆HbO_2_ than RAC (0.03 ± 0.06, *p* = 0.018, partial *η^2^ =* 0.135). Conversely, in the PMC region of CH19, combined RVC and RAC intervention resulted in a decrease in ∆HbO_2_. Overall, RAC showed a more pronounced effect in activating the PFC, while RVC’s influence on S1 activation was slightly superior to RAC. The combined RVC + RAC intervention did not enhance cortical activation levels in patients. Instead, it reduced PMC activation. Based on these findings, we conclude that the improvements in gait associated with RVC and RAC may be attributable to their capacity to enhance activation in specific cortical regions, such as the PFC and S1, as both interventions significantly increased ∆HbO_2_ in these areas compared to the NI group. The differential cortical activation observed between PD-FOG patients and HCs primarily manifests as a significant reduction in ∆HbO_2_ in the S1, M1, and PFC regions in PD-FOG patients. Single-modality interventions with RVC or RAC can significantly elevate ∆HbO_2_ in parts of the PFC and S1, thereby improving cortical activation. However, the RVC + RAC intervention actually reduced ∆HbO_2_ in the PMC, further exacerbating the lower cortical activation observed in PD patients compared to HCs. This may be related to the poor allocation of limited cognitive resources in PD-FOG patients during dual-task mode. Simultaneous use of two different cues may trigger competition for cognitive resources, thereby offsetting the positive effects of a single cue.

### 3.4 The functional connectivity within and between ROIs

#### 3.4.1 Brain network in the PD-FOG NI group compared to HCs

**Figure 7:**
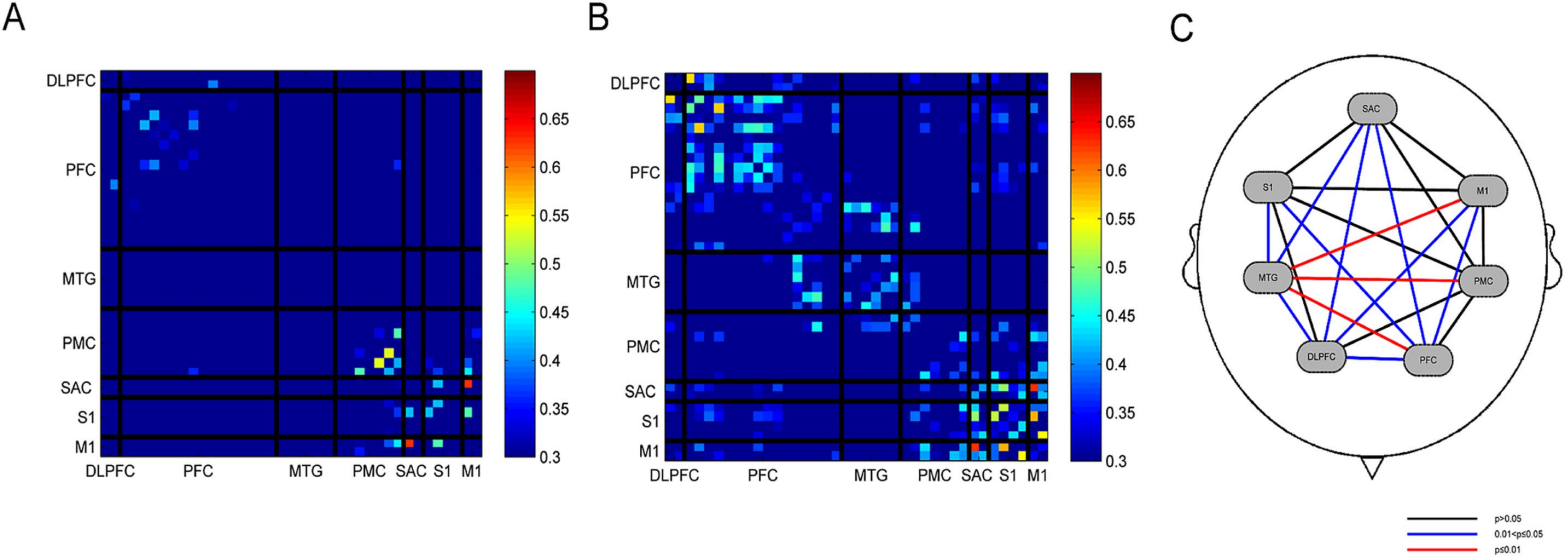
Functional connectivity and ROIs connectivity maps comparing the PD-FOG NI group with HCs in FOG Time. **(A)** FC map of HCs. **(B)** The FC map of the PD-FOG NI group shows slightly higher FC than in HCs. **(C)** Pairwise ROIs connectivity between the PD-FOG NI group and HCs during FOG Time.

To further analyze functional connectivity between the PD-FOG NI group and HCs at the ROI level, we employed Pearson’s correlation analysis to quantify temporal correlations between channels or ROIs, denoted as FC. Figures 7A,B separately display the FC matrices of HCs and PD-FOG NI group based on ROI-level connectivity during the FOG Time, with colors approaching red indicating higher functional connectivity. The FC in HCs is significantly lower than that in the PD-FOG NI group, suggesting increased brain network connectivity in the PD-FOG patients. Figure 7C and Table 5 jointly present the pairwise FC analysis results for different ROIs across both groups. Specifically, Table 5 details the significant pairwise connections among the ROIs, including the PMC, S1, M1, MTG, PFC, DLPFC, and SAC. For example, the S1-MTG connection shows significantly higher FC in the PD-FOG (0.20 ± 0.27) compared to the HCs (0.05 ± 0.13, *p* = 0.013, Cohen’s *d* = 0.704); similarly, the MTG-PFC connection exhibits a higher FC in the PD-FOG (0.22 ± 0.21) than in HCs (0.06 ± 0.11, *p* = 0.001, Cohen’s *d* = 0.941). Overall, these results indicate significant differences in functional connectivity strength across multiple ROI pairs, with the PD-FOG NI group demonstrating markedly higher connectivity than healthy individuals.

**Table 5:** Functional connectivity value of the PD-FOG NI group and HCs in FOG time between different ROIs.

ROI–ROI pair	PD-FOG (*n* = 28)	HCs (*n* = 28)	Statistical value	*p*-value	Cohen’s *d*
PMC-MTG	0.22 ± 0.21	0.05 ± 0.11	3.760	0.001	1.000
S1-MTG	0.20 ± 0.27	0.05 ± 0.13	2.599	0.013	0.704
S1-PFC	0.29 ± 0.22	0.15 ± 0.18	2.442	0.018	0.697
M1-MTG	0.23 ± 0.29	0.01 ± 0.14	3.702	0.001	0.961
M1-PFC	0.28 ± 0.26	0.12 ± 0.15	2.819	0.007	0.748
M1-DLPFC	0.32 ± 0.37	0.10 ± 0.26	2.621	0.011	0.685
MTG-PFC	0.22 ± 0.21	0.06 ± 0.11	3.571	0.001	0.941
MTG-SAC	0.19 ± 0.31	0.02 ± 0.19	2.442	0.019	0.659
MTG-DLPFC	0.23 ± 0.28	0.01 ± 0.22	3.194	0.002	0.873
PFC-SAC	0.28 ± 0.26	0.11 ± 0.18	2.898	0.005	0.762
PFC-DLPFC	0.32 ± 0.27	0.17 ± 0.20	2.392	0.020	0.630
SAC-DLPFC	0.34 ± 0.38	0.10 ± 0.34	2.475	0.016	0.665

The results of brain activation in the two groups indicate that cortical activation in healthy individuals is significantly higher than in the PD-FOG NI group. This may be attributed to degenerative changes in dopaminergic neurons within the basal ganglia-thalamocortical loop in PD-FOG patients, which directly reduce neuronal excitability in key cortical regions, such as the M1 and SMA, manifesting as decreased oxyhemoglobin concentration during task states. Conversely, the PD-FOG exhibits increased FC within brain networks, likely representing a compensatory mechanism in which the brain enhances connectivity between specific ROIs to offset diminished cortical activation and preserve functional integrity. Notably, the ∆HbO_2_ in the S1, M1, and PFC are markedly reduced in PD-FOG patients; however, these regions demonstrate strengthened inter-regional FC at the network level to compensate for this deficit: S1-PFC (*t* = 2.442, *p* = 0.018, Cohen’s *d* = 0.697); M1-PFC (*t* = 2.819, *p* = 0.007, Cohen’s *d* = 0.748). Additionally, the FC strength between other ROIs is significantly elevated in the PD-FOG group, including S1-MTG (*t* = 2.599, *p* = 0.013, Cohen’s *d* = 0.704); M1-MTG (*t* = 3.702, *p* = 0.001, Cohen’s *d* = 0.961); M1-DLPFC (*t* = 2.621, *p* = 0.011, Cohen’s *d* = 0.685), among others (see Table 5 for details). Furthermore, intra-regional connectivity within certain ROIs is also significantly enhanced compared to HCs. As shown in Table 6, these increases in FC within these brain regions are statistically significant: MTG-MTG (*t* = 4.466, *p* < 0.001, Cohen’s *d* = 1.215), PFC-PFC (*t* = 2.656, *p* = 0.010, Cohen’s *d* = 0.697), and SAC-SAC (*t* = 2.943, *p* = 0.005, Cohen’s *d* = 0.790).

**Table 6:** Functional connectivity value of the PD-FOG NI group and HCs in FOG time within ROIs.

ROI–ROI pair	PD-FOG (*n* = 28)	HCs (*n* = 28)	Statistical value	*p-*value	Cohen’s *d*
MTG-MTG	0.37 ± 0.25	0.11 ± 0.17	4.466	<0.001	1.215
PFC-PFC	0.31 ± 0.24	0.17 ± 0.15	2.656	0.010	0.697
SAC-SAC	0.52 ± 0.59	0.13 ± 0.37	2.943	0.005	0.790

#### 3.4.2 Brain network differences among the four PD-FOG subgroups

**Figure 8:**
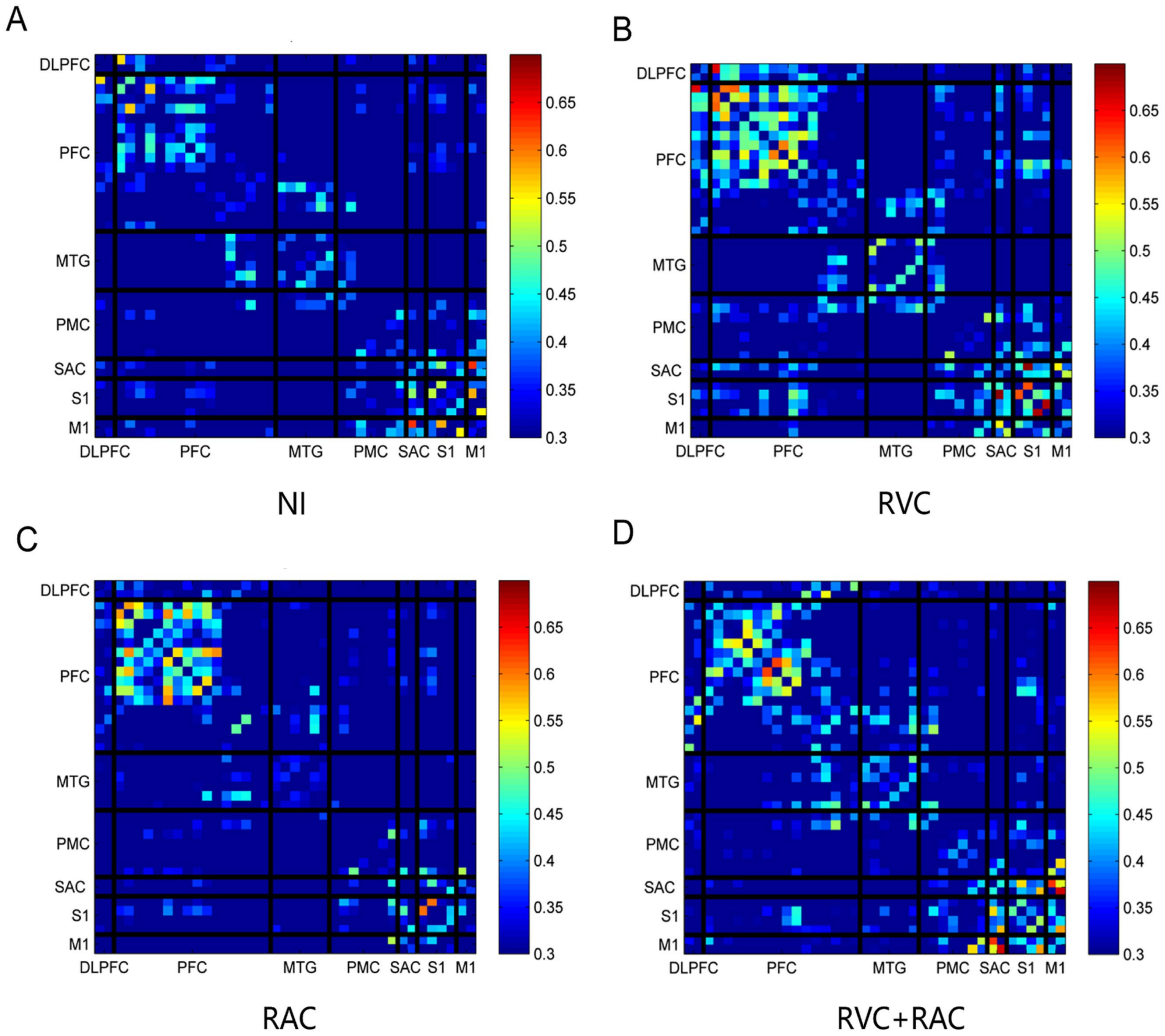
Functional connectivity maps of the four PD-FOG groups across FOG Time. **(A)** FC map of the NI group. **(B)** FC map under RVC intervention. **(C)** FC map under RAC intervention. **(D)** FC map under RVC + RAC intervention.

The FC matrix diagrams of the four PD-FOG groups across the seven ROIs are illustrated in Figure 8. The effects of NI, RVC, RAC, and RVC + RAC intervention on FC predominantly involve brain regions such as the PFC, S1, M1, and PMC. Notably, across different cueing interventions, FC is partially enhanced compared to the NI group. Further statistical analysis reveals that FC based on ROIs division does not show significant pairwise connections; however, channel-level analysis uncovers numerous statistically significant results. Table 7 summarizes 16 channel pairs with meaningful connectivity.

**Table 7:** Functional connectivity value of the PD-FOG four intervention groups in FOG Time between different channels.

CH–CH pair	ROI–ROI pair	NI	RVC	RAC	RVC+RAC	Statistical value	*p*-value	Partial *η^2^*
4–7	PFC-PFC	0.17 ± 0.46	0.28 ± 0.43	0.16 ± 0.36	0.46 ± 0.50	2.956	0.036	0.099
4–43	PFC-PMC	0.19 ± 0.34	0.35 ± 0.47	0.05 ± 0.37	0.29 ± 0.46	3.014	0.034	0.100
9–39	PFC-S1	0.30 ± 0.39	0.45 ± 0.46	0.17 ± 0.46	0.42 ± 0.38	2.767	0.046	0.093
11–22	PFC-PFC	0.20 ± 0.48	0.42 ± 0.39	0.53 ± 0.46	0.43 ± 0.39	3.036	0.033	0.101
11–27	PFC-PFC	0.28 ± 0.53	0.52 ± 0.43	0.68 ± 0.48	0.54 ± 0.44	3.879	0.011	0.126
12–48	PFC-V1	0.17 ± 0.37	0.31 ± 0.41	0.02 ± 0.42	0.04 ± 0.31	3.112	0.030	0.103
14–37	PFC-M1	0.22 ± 0.50	0.06 ± 0.47	0.00 ± 0.44	0.36 ± 0.36	3.397	0.021	0.112
15–23	MTG-PFC	−0.11 ± 0.35	0.08 ± 0.42	0.21 ± 0.44	0.25 ± 0.45	4.119	0.009	0.132
17–36	MTG-PMC	0.17 ± 0.43	0.00 ± 0.41	0.27 ± 0.50	0.34 ± 0.38	2.839	0.042	0.095
17–40	MTG-S1	0.18 ± 0.40	0.07 ± 0.40	0.30 ± 0.43	0.41 ± 0.40	3.150	0.029	0.104
22–25	PFC-PFC	0.14 ± 0.55	0.36 ± 0.50	0.35 ± 0.48	0.63 ± 0.49	3.971	0.010	0.128
23–27	PFC-PFC	0.07 ± 0.46	0.39 ± 0.43	0.38 ± 0.40	0.30 ± 0.39	3.549	0.017	0.116
23–30	PFC-PMC	0.01 ± 0.39	0.24 ± 0.41	0.26 ± 0.41	0.32 ± 0.41	2.914	0.038	0.097
24–42	PFC-PMC	0.16 ± 0.42	0.38 ± 0.47	0.08 ± 0.41	0.21 ± 0.38	2.795	0.044	0.094
34–42	PMC-PMC	0.11 ± 0.44	0.46 ± 0.51	0.24 ± 0.43	0.36 ± 0.39	3.119	0.029	0.104
43–46	PMC-V2	0.05 ± 0.51	0.40 ± 0.37	0.12 ± 0.31	0.24 ± 0.45	3.501	0.018	0.115

V1, primary visual cortex; V2, visual association cortex.

According to Table 7, the RVC intervention significantly increased FC in several channel pairs, including CH23-CH27 (0.07 ± 0.46 vs. 0.39 ± 0.43, *F* = 3.549, *p* = 0.017, partial *η^2^ =* 0.116), CH34-CH42 (0.11 ± 0.44 vs. 0.46 ± 0.51, *F* = 3.119, *p* = 0.029, partial *η^2^ =* 0.104), and CH43-CH46 (0.05 ± 0.51 vs. 0.40 ± 0.37, *F* = 3.501, *p* = 0.018, partial *η^2^ =* 0.115). Since CH23 and CH27 are located in the PFC, CH42 and CH43 are in the PMC, and CH46 is in the V2, it can be concluded that RVC intervention enhances intra-PFC and PMC connectivity and increases FC between PMC and V2. This may represent a core mechanism by which visual cues improve FOG symptoms. Regarding RAC intervention, significant FC increases are observed in channels such as CH11-CH22 (0.20 ± 0.48 vs. 0.53 ± 0.46, *F* = 3.036, *p* = 0.033, partial *η^2^ =* 0.101), CH11-CH27 (0.28 ± 0.53 vs. 0.68 ± 0.48, *F* = 3.879, *p* = 0.011, partial *η^2^ =* 0.126), CH15-CH23 (−0.11 ± 0.35 vs. 0.21 ± 0.44, *F* = 4.119, *p* = 0.009, partial *η^2^ =* 0.132), and CH23-CH27 (0.07 ± 0.46 vs. 0.39 ± 0.40, *F* = 3.549, *p* = 0.017, partial *η^2^ =* 0.116). CH11, CH22, CH23, and CH27 are located in the PFC, while CH15 is situated in the MTG. These findings suggest that RAC stimulation enhances intra-PFC connectivity and PFC-MTG connectivity, potentially serving as a compensatory mechanism to mitigate FOG. For the combined RVC and RAC cue interventions, some channels also exhibit increased FC, including CH15-CH23 (−0.11 ± 0.35 vs. 0.25 ± 0.45, *F* = 4.119, *p* = 0.009, partial *η^2^ =* 0.132), CH22-CH25 (0.14 ± 0.55 vs. 0.63 ± 0.49, *F* = 3.971, *p* = 0.010, partial *η^2^ =* 0.128), and CH23-CH30 (0.01 ± 0.39 vs. 0.32 ± 0.41, *F* = 2.914, *p* = 0.038, partial *η^2^ =* 0.097). CH30 is located in the PMC, while the other channels are situated in the PFC and MTG. The combined intervention primarily modulates FC within the PFC, between the PFC-MTG, and between the PFC-PMC to improve FOG symptoms. Since the different interventions target different ROIs, it is difficult to directly compare their effectiveness in improving brain network connectivity. However, in the channel pair CH23-CH27 (PFC-PFC), both RVC (0.39 ± 0.43, *p* = 0.028) and RAC (0.38 ± 0.40, *p* = 0.033) stimulation significantly increased intra-PFC connectivity compared to the NI group (0.07 ± 0.46), with RVC intervention showing a slightly higher effect.

Table 7 and Supplementary Table 2 further reveal statistically significant differences between every pair of intervention groups. Statistically significant differences in FC were found between the RVC and RAC groups in the following four channel pairs: CH4(PFC)-CH43(PMC) (*p* = 0.030, partial *η^2^ =* 0.100); CH9(PFC)-CH39(S1) (*p* = 0.050, partial *η^2^ =* 0.093); CH12(PFC)-CH48(V1) (*p* = 0.033, partial *η^2^ =* 0.103); CH24(PFC)-CH42(PMC) (*p* = 0.031, partial *η^2^ =* 0.094). These findings indicate that visual and auditory cues elicit distinct FC patterns in the PFC, PMC, S1, and V1 regions during FOG intervention. Considering that RVC enhances connections within the PFC, within the PMC, and between the PFC and visual cortex, while RAC enhances connections within the PFC and between the PFC and auditory cortex, the common mechanism of RVC and RAC interventions is to increase FC within the PFC and between the PFC and the sensory cortex (visual/auditory cortex) required for each cue. The distinction lies in RVC’s greater mobilization of connections within the PMC itself and between the PFC and PMC. The primary FC differences between the RAC and RVC + RAC intervention groups were observed in the CH4(PFC)-CH7(PFC) (*p* = 0.047, partial *η^2^ =* 0.099); CH14(PFC)-CH37(M1) (*p* = 0.024, partial *η^2^ =* 0.112). In contrast, RVC and RVC + RAC intervention showed FC differences primarily in CH17(MTG)-CH36(PMC) (*p* = 0.038, partial *η^2^ =* 0.095); CH17(MTG)-CH40(S1) (*p* = 0.025, partial *η^2^ =* 0.104). This suggests that combined visual–auditory cue interventions may elicit greater activation in regions such as MTG, S1, and M1 than single-modality visual or auditory cues.

### 3.5 The coupling relationship between ΔHbO_2_ and gait performance in PD-FOG

To validate the neural-behavioral association between cortical activation and improvements in gait parameters, this study employed a Linear Mixed-Effects Model (LMM) to analyze the correlation between ΔHbO_2_ in the five significant cortical activation channels (CH19, CH23, CH25, CH27, and CH33) and core gait parameters (gait velocity, stride length). Detailed results are presented in Supplementary Table 3.

#### 3.5.1 Correlation between ΔHbO_2_ and gait velocity

LMM revealed a significant negative main effect of activation level in CH25 (PFC) on gait velocity across all cueing conditions (*B* = −0.548, *F* = 10.292, *p* = 0.002). Notably, although the ANOVA results indicated a general increase in average PFC activation during the intervention task, the LMM revealed an inverse relationship at the individual level: faster velocity was associated with lower PFC activation. This phenomenon suggests that increased PFC activation at the group level may reflect heightened cognitive demands resulting from cue intervention. At the same time, individual differences in performance manifest as variations in PFC resource utilization efficiency. These findings align with previous fNIRS studies (Maidan et al., 2016) showing that complex walking conditions increase PFC engagement in PD patients, and are consistent with research linking PFC activation levels to cognitive demands during walking (Pelicioni et al., 2019). These findings support the neural efficiency interpretation: individuals with superior gait performance rely less on compensatory prefrontal resources to achieve faster gait velocity.

#### 3.5.2 Correlation between ΔHbO_2_ and stride length

Unlike gait velocity, the regulatory mechanism of stride length exhibited distinct interaction effects. Significant interaction effects indicated a pronounced positive interaction between RVC and CH33 (*B* = 2.559, *t* = 2.415, *p* = 0.022), suggesting that under visual cue intervention, increased stride length was driven by elevated cortical oxygenation within this channel. A visual cue intervention that modulates stride length has been shown to activate the frontoparietal executive network, reflecting enhanced visual spatial planning and top-down control demands (Le et al., 2023). Therefore, the positive coefficient in this study indicates that enhanced cortical activity covered by CH33 is significantly correlated with increased stride length under the RVC condition. Conversely, under the RAC condition, CH23 exhibited a significant negative interaction (*B* = −1.868, *t* = −2.306, *p* = 0.026). This result supports the “Bypass/Automaticity” hypothesis (Nombela et al., 2013). Research suggests that auditory rhythms can directly drive motor rhythms via subcortical pathways, such as the reticulospinal tract, thereby reducing reliance on impaired cortical motor control circuits. This negative correlation indicates that patients who successfully utilized auditory rhythms to increase stride length effectively reduced cortical load in the CH23 region, thereby improving gait while enhancing neural processing efficiency.

## 4 Discussion

### 4.1 Cortical activation changes: the targeted activation effect of cues on key ROIs

This study found that, in the absence of intervention, PD-FOG patients exhibited significantly lower ∆HbO_2_ in the S1, M1, and PFC regions compared to HCs, suggesting that cortical hypoactivation in these areas may be a neural substrate underlying FOG episodes. Our findings support previous research indicating that S1, M1, and the PFC play crucial roles in the regulation of normal gait; however, dopaminergic neurodegeneration in PD patients often reduces the excitability of sensorimotor cortices, leading to impairments in gait control (Fatima, 2008; Koenraadt et al., 2014; Maidan et al., 2016; Pelicioni et al., 2019).

In our investigation, we observed that single-cue interventions—RVC and RAC—significantly increased ∆HbO_2_ in the PFC and S1. Notably, RAC elicited a more pronounced PFC activation, while RVC more effectively enhanced S1 activity. Specifically, RVC, through visual inputs such as laser-stripe cues, activates the PFC, likely involving visual-motor integration processes. Prior studies have demonstrated that visual cues activate the prefrontal cortex and visual-related networks (Piramide et al., 2020), with the PFC primarily responsible for motor planning and attentional allocation, and participating in cue-mediated gait regulation. Visual cues are processed through the PFC, aligning with our findings that RVC increases PFC activation. Similarly, auditory cues enhance cortical activation in the temporal and frontal lobes (Nelson and Mayhew, 2025), and our results confirm that RAC strengthens PFC cortical activity. The widespread activation of the PFC via RAC, such as through rhythmic music, may relate to auditory-motor synchronization mechanisms. Auditory rhythm serves as an external cue that synchronizes the motor system, reducing reliance on internal cues, facilitating more rhythmic and fluid movements, and ultimately improving overall gait patterns. Regarding S1, recent Functional Magnetic Resonance Imaging (fMRI) studies have demonstrated that S1 is engaged during visual or auditory stimulation (Bauernfeind et al., 2018), suggesting its role in processing sensory feedback related to visual and auditory stimuli. Our findings that both RVC and RAC increase S1 cortical activation further support S1’s involvement in processing rhythmic sensory cues. Based on this, we conclude that single rhythmic cues (RVC/RAC) can target sensory-related networks (S1) and the frontoparietal network (PFC) to compensate for the cortical hypoactivation in PD-FOG patients.

It is worth noting that the audio-visual combined cue did not enhance activation in the aforementioned regions; instead, it decreased ∆HbO_2_ in the PMC. Studies have shown that PMC activity increases during abnormal gait in PD and that the PMC can compensate for medial prefrontal cortex dysfunction when visual cues are present (Hanakawa et al., 1999; Okuma, 2006). However, our findings indicate that PMC activity diminishes during freezing of gait, with reduced cortical activation. This may be due to the increased neural processing load from multiple sensory inputs, which suppresses PMC activation responsible for motor sequence integration, thereby offsetting the positive effects of single cues. This aligns with behavioral results showing that combined interventions did not produce statistically significant improvements in gait parameters such as stride length and gait velocity, suggesting that multiple cues may induce neural resource competition, ultimately impairing motor control in PD-FOG patients.

To further investigate whether these activation changes correlate with gait improvement, we employed an LMM. Results revealed that increased step length under the RVC condition positively correlated with CH33 (S1) activation. fNIRS studies have demonstrated that walking under visual cues activates the parietal–frontal executive network, reflecting enhanced visuospatial planning and top-down control demands (Le et al., 2023). Our study corroborates this finding. The stride length improvement induced by RVC intervention was closely associated with heightened S1 cortical activation. Notably, stride length improvement under RAC conditions showed a negative correlation with CH23 (PFC) activation. This indicated that RAC-mediated stride improvement is associated with reduced PFC load. Although ANOVA results revealed an overall increase in PFC activation levels during RAC intervention (consistent with heightened executive demands), these group findings align with previous fNIRS studies revealing that complex walking tasks recruit greater PFC resources in PD to compensate for motor deficits (Maidan et al., 2016). They also correspond with research showing PFC oxygenation changes in response to walking-related executive demands (Pelicioni et al., 2019). The LMM in this study revealed inverse coupling between RAC and PFC cortical activation at the individual level, supporting the “Bypass/Automaticity” theory. This theory posits that RAC promotes movement through subcortical synchronization, thereby reducing cortical motor control demands (Nombela et al., 2013). Thus, patients who exhibited increased stride length under RAC conditions demonstrated lower PFC load, consistent with a more automated gait control pattern.

### 4.2 Neural network reconfiguration: the optimization of FC based on cue specificity

Our analysis of FC revealed that, in the absence of intervention, patients with PD-FOG exhibit significantly increased FC between multiple brain regions (such as PFC-SAC, S1-PFC, M1-PFC) compared to HCs, consistent with the findings of Wang et al. (2024), suggesting that enhanced brain network connectivity may serve as a compensatory mechanism to offset localized cortical activation deficits. Feng et al. (2023) further demonstrated that increased intra-cortical FC in PD-FOG patients may activate indirect motor networks, which, in conjunction with direct motor pathways, facilitate the control of simple tasks. Based on this, we posit that PD-FOG patients compensate for cortical hypoactivation through increased FC within the indirect motor network, thereby mitigating functional impairments.

Following cue-based interventions, RVC and RAC reconstruct brain networks via distinct patterns. Resting-state fMRI studies indicate that FOG is associated with decreased FC within the sensorimotor network (notably the PMC), the default mode network (particularly the prefrontal and parietal regions), and the visual association network (including the occipital cortex) (Canu et al., 2015). Interestingly, our results show that after RVC intervention, FC is primarily enhanced within the PFC and PMC, as well as between the PMC and V2. This suggests that the improvement in FOG with rhythmic visual cues may be mediated by neural mechanisms involving these regions. Visual cue regulation of gait relies on the coordinated involvement of the PMC, V2, and PFC: visual signals processed in V2 are relayed to the PFC, which integrates visual information and modulates motor output via the PMC, forming a closed loop of “visual input—motor planning—execution” for movement control. The PFC is central to goal-directed motor control, integrating visual information with motor plans, indicating that RVC enhances internal PFC connectivity, thereby strengthening attentional allocation to visual cues and motor goal planning. This supports a mechanism of transitioning from habitual, reliance-based motor control to goal-oriented control, ultimately improving freezing symptoms. Additionally, increased intra-PMC and PMC-V2 connectivity are associated with visual regulation mechanisms; V2 processes visual information, while PMC plays a crucial role in motor programming and modulation, especially for externally cued movements (Wessel et al., 1997). The observed increase in FC between PMC and specific motor-related regions in PD patients has been regarded as a compensatory response to motor deficits (Hao et al., 2020), aligning with the finding that RVC enhances PMC-V2 connectivity, thereby alleviating freezing. RVC also increases FC within the default mode network’s prefrontal regions and within the sensorimotor network’s PMC, partially compensating for gait abnormalities caused by resource deficits (Navarro et al., 2009; Steidel et al., 2021). Our results demonstrate that RVC enhances FC within the PMC and PFC, and between the PMC and V2, which may partially compensate for gait disturbances, such as freezing, resulting from cortical hypoactivation in PD-FOG patients.

Generic auditory stimulation can facilitate motor activation patterns by enhancing connectivity between the frontal lobe and temporal regions (Koshimori and Thaut, 2023), which aligns with our findings that RAC primarily strengthens intra-PFC connections and the connectivity between PFC and MTG. The MTG is involved in auditory rhythm perception and processing, while the PFC integrates auditory cues with motor functions. RAC promotes its synergistic interaction by increasing connectivity between the PFC and MTG, thereby optimizing attention allocation to auditory cues in patients with freezing of gait and enhancing motor planning. Utilizing auditory rhythm to synchronize movement reduces reliance on internal cues, supporting “entrainment” movement patterns, which improve overall motor function and gait performance in freezing patients.

The combined RVC and RAC intervention primarily enhances functional connectivity within the PFC itself, as well as PFC-MTG and PFC-PMC. However, these strengthened connections are predominantly PFC-related, reflecting the PFC’s role in cognitive regulation. This suggests that the combined cues require greater cognitive resource engagement, which may explain their limited direct impact on gait improvement.

### 4.3 Clinical application: integrating the neural mechanisms of cues with clinical practice

Compared to other cutting-edge interventions for FOG [such as Deep Brain Stimulation (DBS)], non-invasive brain stimulation including Transcranial Direct Current Stimulation (tDCS), and wearable sensor-based adaptive cueing systems, our cue-based strategy presents a distinct neural modulation profile. For instance, DBS improves FOG by modulating pathological beta oscillations in the subthalamic nucleus (Tinkhauser et al., 2017). Non-invasive stimulation therapies have also been explored in PD-FOG; for instance, multi-target tDCS has been validated to modulate the motor-cognitive network in freezing patients (Dagan et al., 2018). However, these cutting-edge neuromodulation techniques require specialized equipment and clinical facilities. In contrast, rhythmic cueing interventions require no invasive procedures or large specialized equipment, directly activating cortical and subcortical networks involved in motor planning and sensory integration. Moreover, more state-of-the-art methods, such as emerging closed-loop (adaptive) cueing systems (Mancini et al., 2018), can trigger precise interventions by detecting freezing episodes in real time. But their complex algorithms and high hardware costs may limit widespread home adoption. Our fNIRS research indicates that open-loop rhythmic cues remain a reliable alternative, providing stable external sensory feedback to the motor system. Crucially, beyond gait improvements, our fNIRS findings provide neurophysiological support for the mechanism of visual/auditory cueing. Under RVC conditions, increased stride length positively correlated with heightened activation in sensorimotor integration areas (e.g., S1), suggesting an enhanced reliance on external spatial templates. Conversely, under RAC cueing, stride length increased, accompanied by reduced PFC load, indicating a shift toward more automated cue control patterns. This neurophysiological difference provides a precise explanation for the cue-specific responses observed in PD-FOG patients and identifies potential neural targets for personalized rehabilitation therapy.

Regarding the application of rhythmic cues for precision rehabilitation in PD-FOG patients, clinicians should select appropriate cueing strategies based on individual patient characteristics rather than adopting a one-size-fits-all approach. LMM results demonstrated that stride length improvement under RVC was associated with enhanced compensatory activation in sensorimotor integration areas (e.g., S1). Thus, visual cues are more suitable for patients with better executive function and cognition, who can utilize RVC to bypass impaired neural circuits and achieve gait improvement through heightened cortical activation in sensorimotor integration regions (e.g., S1). The improvement in stride length under RAC correlated with reduced PFC activation, which indicated that patients require fewer cognitive resources to achieve enhanced stride length. For patients with relatively poorer cognitive function, RAC may be a more suitable strategy. These promoted gait through subcortical synchronization mechanisms without excessive consumption of executive function resources. However, combined RVC and RAC intervention should be used with caution, as no gait improvement was observed under this condition, and multiple cues may trigger cognitive resource competition in PD-FOG patients. Simultaneous use of both stimuli may exceed the limited attentional resources of PD-FOG individuals, potentially negating the effects of either single cue. Future selection of cueing protocols should be preceded by brief cognitive screening to determine the most appropriate cueing strategy for each individual.

To better apply cue prompts in clinical settings, concurrent gait monitoring is necessary to observe their effectiveness. Standard gait analysis heavily relies on optical motion capture systems. Such equipment requires dedicated laboratory space and incurs high maintenance costs. These limitations hinder its application in primary care facilities or community hospitals. In contrast, the Dynamic Gait and Posture Analysis System employed in this study offers superior portability. By embedding sensors directly into insoles, it enables simultaneous gait data collection during walking without requiring additional auxiliary facilities, significantly enhancing clinical feasibility. Data acquisition is also not restricted by location, with results clearly visualized, facilitating application in restricted environments like community hospitals. Rhythmic cueing offers advantages over other complex neurotechnologies (e.g., DBS, tDCS), including lower cost, portability, and ease of operation, enabling flexible application in hospital wards or home rehabilitation settings. Regarding fNIRS equipment, its use in this study was solely to explore the cortical mechanisms underlying rhythmic cue (RVC, RAC, and RVC + RAC) interventions on gait. It is not recommended for routine clinical treatment. fNIRS results confirm that rhythmic cues effectively modulate cortical activity. We recommend integrating the Dynamic Gait and Posture Analysis System with cue interventions for gait rehabilitation in PD patients. This approach is simple and easy to implement, requiring minimal space and equipment, making it equally suitable for healthcare institutions with limited resources.

## 5 Limitations

This paper still has several limitations. Firstly, we did not recruit PD patients without FOG to compare the cortical mechanisms between freezing and non-freezing patients, primarily due to time constraints, resulting in missing data that limit insights into the fundamental mechanisms of freezing of gait. Our research team plans to follow up by including this patient subgroup to supplement this gap. Secondly, as fNIRS only monitors cortical activity, it cannot assess functional changes in deeper brain structures such as the basal ganglia. Subsequent studies could integrate additional neuroimaging techniques, such as fMRI and EEG, to more comprehensively explore subcortical mechanisms. Thirdly, this study examined brain network connectivity solely through functional correlations between ROIs, lacking analyses of directional influences or causal relationships. Subsequent research should focus on effective connectivity to better understand causal interactions and thereby elucidate the mechanisms underlying FOG. Fourthly, in the experimental paradigm’s FOG Time, we induced freezing episodes through narrow passages and turning maneuvers. However, these induction methods may also alter cortical oxygenation and functional connectivity in PD-FOG patients. This study did not exclude these confounding factors, thereby providing a direction for future research to refine studies of freezing populations. Fifthly, regarding the metrics for gait assessment, we exclusively selected fundamental variables such as stride length, cadence, and gait velocity. Although this data acquisition system can accurately identify spatiotemporal gait parameters, it cannot automatically recognize or capture the turning phase or gait patterns during freezing episodes in PD-FOG patients. Thus, we did not incorporate metrics that better reflect the complexity of freezing, such as stride variability, double support time, or step length asymmetry. This decision somewhat compromised the sensitivity in detecting statistical improvements in freezing phenomena. Future research on gait freezing should consider integrating variables closely related to freezing, such as variability and asymmetry, alongside fundamental variables to enhance the persuasiveness of experimental findings. Sixthly, the clinical assessments of PD-FOG patients did not include evaluations of psychological states such as depression and anxiety, which may influence cortical activity and should be considered in future assessments. Seventhly, regarding cue-synchrony in subjects, we lack objective metrics for quantitatively measuring participants’ gait synchronization with external cues. Currently, compliance with experimental requirements is monitored solely by clinical professionals, a practice that may overlook the possibility that subjects’ gait may not fully match visual cues or rhythmic patterns. Future studies should employ motion capture systems or pressure-sensitive insoles to provide objective metrics of cue-synchrony performance, thereby addressing this limitation and strengthening the evidence for cue-induced gait modifications.

## 6 Conclusion

This study confirms that single rhythmic cues (RVC or RAC) significantly improve stride length and gait velocity-related gait parameters in PD-FOG patients. RVC is more effective than RAC at increasing stride length and gait velocity. Combined stimulation may fail to produce meaningful gait changes due to insufficient cognitive resource allocation caused by the demands of multiple tasks. At the cortical activation level, both RVC and RAC significantly enhance ∆HbO_2_ in the PFC and S1, these two cues influence gait changes through distinct mechanisms. However, the combined intervention reduces PMC activation, possibly because the dual cues increase cognitive load, thereby hindering the effective activation of relevant cortical areas. This aligns with findings that the audio-visual combined cue does not significantly improve gait parameters in PD-FOG patients. Regarding brain network connectivity, cue interventions redirect neural focus toward key ROIs. For instance, RVC targets activation within the PFC and PMC and optimizes visual-motor network connectivity. At the same time, RAC enhances connectivity between the PFC and MTG, as well as the PFC’s internal connections. The strengthened connections among these critical networks offer new insights into how cueing strategies can improve gait in patients with freezing. These findings also provide a neurophysiological basis for non-pharmacological treatments of PD-FOG patients, supporting the clinical application of single rhythmic cues for precise intervention.

## Data Availability

The original contributions presented in the study are included in the article/Supplementary material, further inquiries can be directed to the corresponding author.
