# Supplementary material for: The mechanism of external cueing interventions in improving freezing of gait in Parkinson’s disease: an fNIRS study
Source: Front Aging Neurosci. 2026 Mar 20;18:1776882. doi: 10.3389/fnagi.2026.1776882 (PMC13046713; doi:10.3389/fnagi.2026.1776882)
Supplement: Supplementary file 1 [file Data_Sheet_1.docx]

Supplementary Material

# Supplementary Tables

**Supplementary Table 1**: Gait parameters of the PD-FOG four intervention groups in FOG Time.

| **Gait parameters** | **NI** | **RVC** | **RAC** | **RVC+RAC** | **Statistical value** | ***p*-value** | **partial *η²*** | ***p*-value (*post-hoc*)** | | |
| --- | --- | --- | --- | --- | --- | --- | --- | --- | --- | --- |
|  |  |  |  |  |  |  |  | **NI vs RVC** | **NI vs RAC** | **NI vs RVC+RAC** |
| Stride length (cm) | 50.14±15.42 | 66.54±21.28 | 60.79±14.33 | 51.82±16.74 | 7.185 | .001 | 0.210 | < .001 | .003 | 0.694 |
| Gait velocity (m/s) | 0.43±0.21 | 0.66±0.17 | 0.55±0.12 | 0.47±0.16 | 12.987 | .001 | 0.325 | < .001 | .005 | 0.363 |
| Cadence (steps/min) | 104.88±18.11 | 110.45±18.82 | 110.98±16.31 | 103.42±12.91 | 2.131 | 0.111 | 0.073 | / | / | / |

**Supplementary Table 2:** *Post-hoc* examination of functional connectivity among the PD-FOG four intervention groups in FOG Time

| **CH-CH pair** | **ROI-ROI pair** | ***p*-value (*post-hoc*)** | | | | | |
| --- | --- | --- | --- | --- | --- | --- | --- |
|  |  | **NI vs RVC** | **NI vs RAC** | **NI vs RVC+RAC** | **RVC vs RAC** | **RVC vs RVC+RAC** | **RAC vs RVC+RAC** |
| 4-7 | PFC-PFC | 0.734 | 1.000 | 0.056 | 0.689 | 0.413 | 0.047 |
| 4-43 | PFC-PMC | 0.488 | 0.525 | 0.820 | 0.030 | 0.957 | 0.129 |
| 9-39 | PFC-S1 | 0.469 | 0.637 | 0.713 | 0.050 | 0.985 | 0.130 |
| 11-22 | PFC-PFC | 0.199 | 0.021 | 0.266 | 0.792 | 0.999 | 0.736 |
| 11-27 | PFC-PFC | 0.141 | .007 | 0.151 | 0.704 | 1.000 | 0.727 |
| 12-48 | PFC-V1 | 0.551 | 0.456 | 0.674 | 0.033 | 0.091 | 0.992 |
| 14-37 | PFC-M1 | 0.534 | 0.235 | 0.728 | 0.949 | 0.095 | 0.024 |
| 15-23 | MTG-PFC | 0.349 | 0.022 | 0.014 | 0.592 | 0.454 | 0.994 |
| 17-36 | MTG-PMC | 0.434 | 0.877 | 0.598 | 0.110 | 0.038 | 0.949 |
| 17-40 | MTG-S1 | 0.699 | 0.723 | 0.263 | 0.151 | 0.025 | 0.840 |
| 22-25 | PFC-PFC | 0.375 | 0.409 | 0.005 | 1.000 | 0.242 | 0.206 |
| 23-27 | PFC-PFC | 0.028 | 0.033 | 0.170 | 1.000 | 0.920 | 0.945 |
| 23-30 | PFC-PMC | 0.188 | 0.111 | 0.039 | 0.994 | 0.861 | 0.951 |
| 24-42 | PFC-PMC | 0.165 | 0.885 | 0.981 | 0.031 | 0.356 | 0.696 |
| 34-42 | PMC-PMC | 0.027 | 0.710 | 0.155 | 0.286 | 0.930 | 0.690 |
| 43-46 | PMC-V2 | 0.014 | 0.875 | 0.337 | 0.117 | 0.570 | 0.797 |

**Supplementary Table 3:** Linear mixed effects model results for ΔHbO2 and gait performance in PD-FOG

| **Gait parameters** | **Predictor** | ***B*** (Estimate) | **Statistic value** | ***p***-value | **95% CI** |
| --- | --- | --- | --- | --- | --- |
| Gait velocity (m/s) | Intercept | 0.520 | 123.060 | <0.001 | [0.36, 0.68] |
|  | CH19 | −0.01 | 0.360 | 0.551 | [−1.02, 1.00] |
|  | CH23 | 1.000 | 6.490 | 0.013 | [−0.43, 2.44] |
|  | CH25 | −0.548 | 10.292 | 0.002 | [−1.60, −0.50] |
|  | CH27 | 0.540 | 0.030 | 0.859 | [−0.57, 1.65] |
|  | CH33 | 0.050 | 1.500 | 0.224 | [−1.24, 1.33] |
| Stride length | Intercept | 0.570 | 377.750 | <0.001 | [0.47, 0.66] |
|  | CH19 | 0.110 | 0.050 | 0.826 | [−0.62, 0.84] |
|  | CH23 | 0.610 | 0.400 | 0.531 | [−0.44, 1.66] |
|  | CH25 | 0.190 | 0.170 | 0.679 | [−0.57, 0.95] |
|  | CH27 | 0.430 | 0.960 | 0.331 | [−0.38, 1.25] |
|  | CH33 | −0.82 | 0.580 | 0.449 | [−1.76, 0.12] |
|  | RVC × CH33 | 2.559 | 2.415 | 0.022 | [0.40, 4.72] |
|  | RAC × CH23 | −1.868 | −2.306 | 0.026 | [−3.50, −0.24] |

Abbreviations: *B*, Unstandardized Coefficient (Estimate); CI, Confidence Interval.
